# Seasonal Effect on Tree Species Classification in an Urban Environment Using Hyperspectral Data, LiDAR, and an Object-Oriented Approach

**DOI:** 10.3390/s8053020

**Published:** 2008-05-06

**Authors:** Matthew Voss, Ramanathan Sugumaran

**Affiliations:** GeoTREE Center, 205 Innovative Teaching & Technology Center, Cedar Falls, Iowa, 50614-0406, USA; E-mail: sugu@uni.edu

**Keywords:** remote sensing, object oriented, hyperspectral, LiDAR, tree species, urban

## Abstract

The objective of the current study was to analyze the seasonal effect on differentiating tree species in an urban environment using multi-temporal hyperspectral data, Light Detection And Ranging (LiDAR) data, and a tree species database collected from the field. Two Airborne Imaging Spectrometer for Applications (AISA) hyperspectral images were collected, covering the Summer and Fall seasons. In order to make both datasets spatially and spectrally compatible, several preprocessing steps, including band reduction and a spatial degradation, were performed. An object-oriented classification was performed on both images using training data collected randomly from the tree species database. The seven dominant tree species (*Gleditsia triacanthos*, *Acer saccharum*, *Tilia Americana, Quercus palustris*, *Pinus strobus* and *Picea glauca*) were used in the classification. The results from this analysis did not show any major difference in overall accuracy between the two seasons. Overall accuracy was approximately 57% for the Summer dataset and 56% for the Fall dataset. However, the Fall dataset provided more consistent results for all tree species while the Summer dataset had a few higher individual class accuracies. Further, adding LiDAR into the classification improved the results by 19% for both fall and summer. This is mainly due to the removal of shadow effect and the addition of elevation data to separate low and high vegetation.

## 1. Introduction

Tree species maps are important for several reasons. Municipal governments use land cover maps for conservation, such as the preservation of a particular tree species [1]. Growing cities also have a desire to control development near greenbelt areas [2]. Tree species maps can also be used by conservationists hoping to protect the favored nesting place of a particular species of bird [3]. Thus, there is demand for accurate and up-to-date land cover maps. Remote sensing approaches have proven to be valuable in developing land cover maps compared to traditional methods [5, 4]

There is a considerable amount of literature regarding the identification and classification of tree species utilizing airborne or space-borne imagery using numerous classification methods. Generally, tree species identification using remote sensing data depends upon spatial, spectral and temporal resolution. In addition, several authors discuss the importance of different classification algorithms and supplementary data such as LiDAR for the identification of tree species.

The first major use of digital imagery and machine processing was to map vegetation health a year after the corn leaf blight in 1970 [6]. The launch of Landsat in 1972 began a serious investigation into the capabilities of remote sensing for vegetation management. In 1978, Kan and Weber released their study on mapping forests and rangelands using Landsat. They found they could separate hardwood forest, softwood forest and grasslands with 70% accuracy [7].

Meyer, Staenz, and Itten used color-infrared film to image two areas of the Swiss Plateau and were able to classify 5 classes of trees with 80% accuracy [8]. In another study, Cypress and Tupelo trees were mapped utilizing moderate spatial resolution Landsat TM imagery in an effort to develop a method of locating wetland areas for more effective land management [9]. Higher spatial resolution imageries were also used by several authors for tree species identification [11, 10]. Carleer and Wolff attempted an analysis of tree species in a Belgian forest using a high-resolution IKONOS image [12]. They suggest that forest tree mapping requires higher spatial and spectral resolution.

Combinations of different date and spatial resolution multi-spectral images have been used for species classification [1]. They found that the images taken in September were most useful in identification of tree species and 1m spatial resolution is optimal for reducing the shadow effects in between the trees in Columbia, Missouri. Fall imagery appeared to provide the most information for species identification while spring leaf-out imagery was next best in terms of species identification [13].

Spectral resolution is also a significant factor in determining overall classification accuracy. Comparatively few authors have used hyperspectral imagery for tree species identification. Researchers compared four satellite-based sensors – the Hyperion hyperspectral scanner, IKONOS, Landsat ETM+ and the ALI multispectral scanner – and used them to identify species in an African rainforest [14]. They found the hyperspectral results to be 45 to 52% better than multispectral imagery in classifying land cover. AVIRIS data has been used to identify tree types for urban mapping in the city of Modesto, California [15]. Results indicate that high-resolution, hyperspectral data is an excellent tool for species identification. le Maire, Francios and Dufrene researched methodologies for differentiating tree species [16]. In particular, they review various ratios and band combinations that have been implemented by other researchers. This research determined that hyperspectral data does improve classification accuracy. Greiwe and Ehlers used a high-resolution, three-dimensional sensor in combination with 128 bands of HyMap data to classify the city of Osnabrueck in Germany [17].

Several researchers have also reported the importance of advanced classification methods such as subpixel classification, Classification And Regression Tree (CART) [1], object–oriented and support vector machines for the mapping of tree species. These methods generally rely on more advanced data analysis methods borrowed from the field of statistics. Subpixel classification has been found to improve accuracy by as much as 18 percent over traditional classification methods such as minimum distance [9]. Linear Discriminant Analysis is a means of statistical analysis that has provided more accurate results than traditional methods of classification, when identifying tree species with hyperspectral imagery [18].

In 1980 David Landgrebe published an article in which he described a spatial-spectral classification method [19]. This method is now known as object-oriented classification. Recently, object-oriented classification methods have become more accessible to researchers due to software such as eCognition Professional (Definiens AG) and Feature Analyst (Visual Learning Systems Inc.). One of the strengths of object-oriented classification is that a pixel no longer represents a single object, but rather a component of an object [20]. Segments also have shape, location and texture components that can be used for classification. Pixel-based classifiers have difficulty dealing with the spectral variations in tree crowns [21]. Object-oriented classifiers allow user's to treat a crown as one object. Kristof, Csato and Ritter used 1m panchromatic and 4m multispectral IKONOS imagery and an object oriented classification scheme to classify a forest in Hungary [22]. This was done after poor results were obtained using pixel-based methods. eCognition was used to classify a mountainous region in the Czech Republic [23]. The classification of conifers obtained more than 90 percent accuracy. Object-oriented classification has been used to identify tree species in forests in the northeast United States [10]. It was again applied to a mountainous forested region, this time in Japan [11]. Researchers have started to combine several advanced classification methods such as decision trees and object oriented with good results [24].

Elevation data have been incorporated as a data source in many studies. Iovan, Boldo and Cord used a high resolution Digital Elevation Model (DEM) to compliment high resolution color infrared image to classify vegetation in an urban setting. Heights were used to separate grass from shrubs and trees as well to delineate tree crowns [25]. Commonly, LiDAR is the source for this elevation data. It has been used with hyperspectral AISA data to determine which frequencies provide the best species separation [26]. LiDAR and HYDICE multispectral imagery were used for classification of tree species in a tropical environment [18].

LiDAR has also been used to develop a method of identifying pine and spruce trees [27]. Using data collected over a Norwegian forest, researchers were able to correctly classify pine and spruce trees with 95% accuracy by using the differences in shape between the two tree types. LiDAR and very high resolution multispectral imagery were used to map tree species in a wildlife refuge in Mississippi [28]. Laser-based altimetry data and multispectral IKONOS imagery were used to estimate pine tree coverage in a forest [29]. One of the principle discoveries of this study was that LiDAR may be an effective method of estimating Leaf Area Index. Researchers have also used LiDAR to determine relations between branch/stem structure and species [30].

Much of the current research with LiDAR explores its usefulness in estimating physical characteristics of trees. These characteristics may include heights, basal areas and volumes of pine and spruce trees [31] and leaf area index in loblolly pines [32]. It has also been used to create Digital Canopy Models in densely forested areas [33]. LiDAR has been used to develop a methodology to segment individual trees in a forest, and these segments were used to estimate heights and crown diameters [34]. LiDAR has been used to define tree clusters (rather than individual tree segments) which were used to estimate density and biomass [35], and researchers have segmented LiDAR canopy data to determine tree stem locations [36]. Another study has used LiDAR to develop volumetric models that allow for more accurate approximation of biomass [37].

In general, the literature indicates that best classification accuracies can be obtained using hyperspectral data, LiDAR and an advanced classification methodology. The goal of this research was to determine the affect of season on tree species classification in an urban environment using hyperspectral imagery and object-based classification. In addition, this research explored the fusion of LiDAR data with imagery for improving classification accuracy.

## 2. Study Area and Data

The University of Northern Iowa campus was selected as a study area. The campus covers approximately 49 hectares and is located in Cedar Falls, Iowa, USA. It is relatively flat, with a slight decrease in elevation toward the southern edge of the study area. This study area was chosen for several reasons. First, the researchers have knowledge of the study area. Second, the campus has a wide variety of mature trees, many of which are separated from other trees by an expanse of grass, reducing the possibility of confusing samples with another species. The dominant deciduous trees are *Gleditsia triacanthos*, *Acer saccharum*, *Tilia americana* and *Quercus palustris*; the dominant evergreens are *Pinus strobus* and *Picea glauca*. Third, the University's planning department has information about each of the trees on the campus, which saves researchers the time it would take to identify them.

This study benefited from having two hyperspectral datasets from different data collecting campaigns. The first dataset was collected on July 31, 2004 with an AISA (Airborne Imaging Spectrometer for Applications) hyperspectral sensor. This image contains data from 400nm to 850nm with 2.0m spatial resolution. The second hyperspectral dataset was collected in October 2006 by an improved version of the AISA sensor called the Eagle. This sensor captured 63 bands from 400 to 970nm and 1m spatial resolution. Additional data included a LiDAR dataset which was collect in April 2006 by Sanborn Mapping Company Inc. with a Leica ALS50 with a maximum sampling rate of 83 kHz. These were delivered in nine-1 km^2^ tiles with 1m posting density. Finally, a tree database containing all trees and shrubbery on campus was used to develop both training and reference datasets.

## 3. Methodology

### 3.1 Image processing

Before any image processing was done, both hyperspectral datasets were geometrically referenced to the LiDAR imagery using ENVI 4.4 (ITT). For the Fall image, 30 ground control points were selected. There were 39 ground control points for the Summer image. In order to make both datasets compatible spatially and spectrally, the Fall image was spatially degraded to 2m resolution, and the last 14 bands of the image (from approximately 850 to 970nm) were removed and every second band was removed to produce an image with 25 bands. This provided an image that is equal to the Summer image in terms of both spatial and spectral resolution. The imagery was then processed using a forward Minimum Noise Fraction transform to remove noise from the dataset. The decision to do this was made after performing a quick Linear Discriminant Analysis of samples which indicated a high classification error rate (.35) with raw imagery. The MNF data resulted in a lower classification error rate (.25).

The LiDAR dataset was processed from its native .las format to a raster elevation and intensity dataset with 1m spatial resolution using LiDAR Analyst v.4.2. Bare earth elevations were subtracted from the first return elevation to create a relative altitude layer where the new elevation of the ground was ideally 0m. The LiDAR data were spatially degraded to 2m to match the resolution of the other imagery in the study. The original posting of the LiDAR was 0.6m. However, using a DEM created at that resolution created complex issues of scale which we most easily solved by degrading the LiDAR resolution.

### 3.2 Training and Reference Data

One of the unique aspects of this project was having a complete reference for all the vegetation in the study area in the form of an ESRI shapefile. Developing a training and reference data set can be a costly, time-consuming process. These costs were absorbed by the Facilities Planning department when they created this database. Training and reference data were derived from this shapefile. Before any analysis was performed, the shapefile was clipped to the image with the smallest extent (the Summer image). Using a frequency plot, the 7 most common trees in the study area were selected. These trees were then selected from the main database into 7 separate shapefiles for further analysis. To ensure the trees selected from this database were out of shadows, trees within 10m of a building were removed from the dataset. In addition, smaller trees were removed from the database by selecting out those trees with a crown diameter less than 4.5m.

With the database refined, the samples and references were selected. A random sampling strategy was utilized for the selection of training sample. Following the database preparation, the least numerous tree species had 50 locations in the study area. Thirty training samples and 20 reference samples were selected. A previous rule of thumb for developing training sets has been to have 30n samples where n is the number of bands, although it has been found that similar accuracies could be attained with 2n to 4n samples [38]. In our case, 30n samples was impossible as there are not that many trees, particularly in the case of the less common species. Random points were selected by creating a simple spreadsheet with shapefile ID numbers in one column and a randomly generated number in the other. The fields were then sorted by the random number and the first 30 ID numbers were selected out of the shapefile to create a new file. These 30 points were then removed from the original database and 20 more points were selected randomly for a reference set if there were more than 20 points remaining. The individual training shapefiles were then merged into a larger field, and converted into a raster GRID format for use with object-oriented classification. This process was repeated for the reference shapefiles.

### 3.3 Segmentation

Segmentation is one of the most critical aspects of object-based classification. Segment size can have a significant effect on classification accuracy. The primary setting that determines segment size is the scale parameter. Secondary settings are shape and compactness. There does not appear to be any tool to determine the ideal parameters, so a trial and error method was employed. The initial scale parameter setting was determined by visual inspection. It is obvious that the segments should not be so large as to encompass multiple trees. Ten was determined to be the maximum scale parameter (and subsequent analysis confirmed this). After that, multiple classifications were performed at different scale parameters by creating a simple script to perform repetitive tasks. This script loaded the sample dataset, created samples from it, classified the imagery and then loaded the reference dataset.

**Figure 1.**
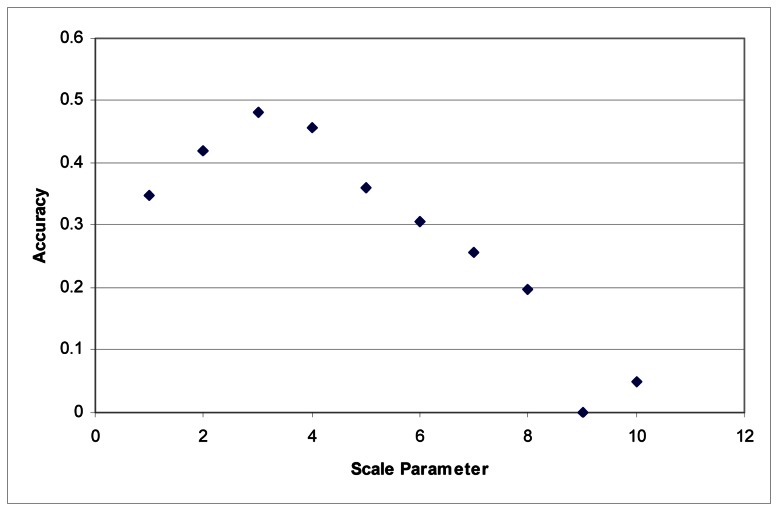
Segmentation scale parameter vs. accuracy without LiDAR for the Summer dataset.

**Figure 2.**
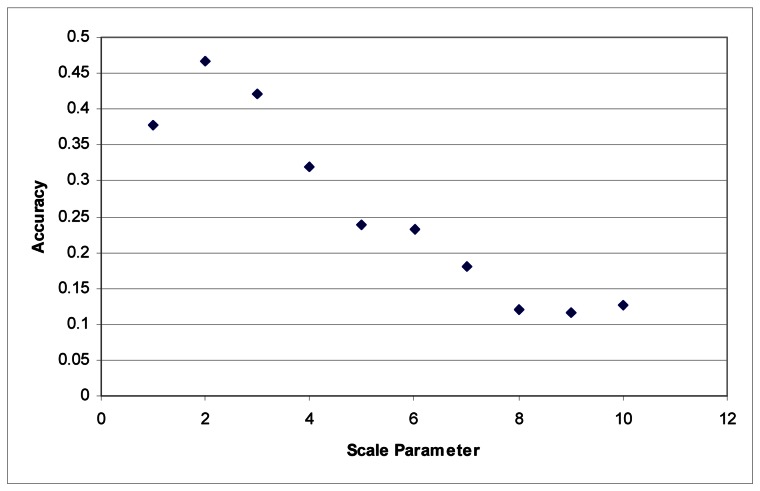
Segmentation scale parameter vs. accuracy without LiDAR for the Fall dataset.

These tests were first performed using the imagery to develop the segments. Maximum accuracy for the Summer dataset was obtained at a scale parameter of 3 (Figure 1). With the Fall dataset, the ideal scale parameter was achieved at a scale of 2 (Figure 2).

Additionally, accuracies decline quite rapidly. Above a segmentation scale parameter of 10 accuracies quickly drop to 0. Higher scale parameters result in larger segments, and at these scales, many of the classes are no longer classified because each segment contains samples for several classes. This is responsible for the fairly rapid decline in overall accuracy in these tests.

**Figure 3.**
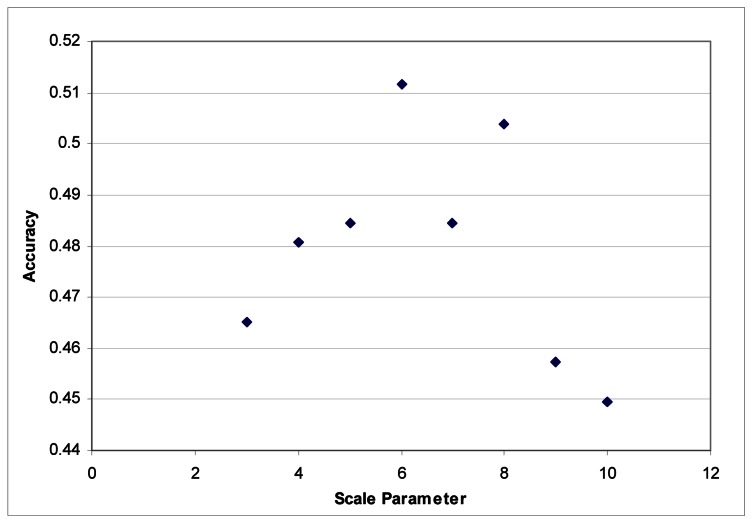
Segmentation scale parameter vs. accuracy for the Summer dataset.

**Figure 4.**
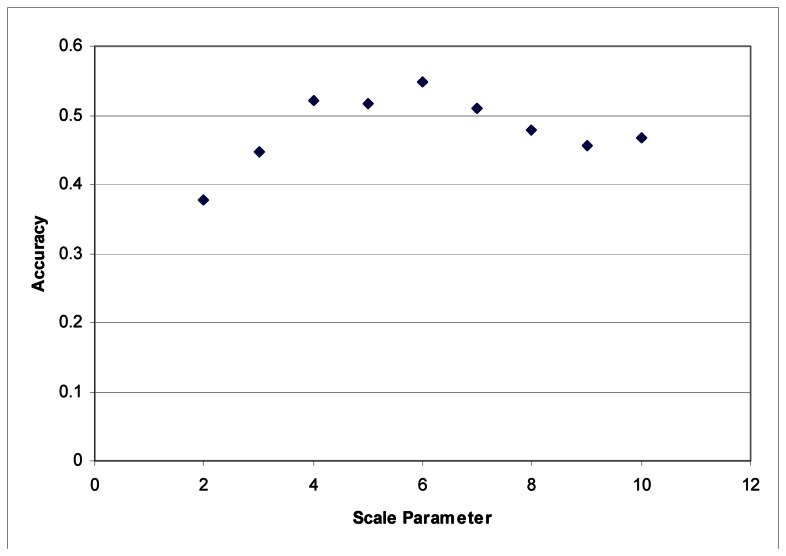
Segmentation scale parameter vs. accuracy for the Fall dataset.

These segmentation tests were performed using the LiDAR to generate segments. The images were segmented based on the LiDAR intensity and the customized elevation layer without any influence from the hyperspectral data. It was found that it was difficult to develop good segments based on imagery as the shadows created tree segments that included the shadows lying on the grass. LiDAR data does not contain any shadows, and in this instance, elevation is an ideal criterion for separating classes. Maximum accuracies were obtained with a scale parameter of 6 for each dataset (Figures 3 and 4).

These tests were also performed on the shape and smoothness-compactness parameters. These final parameters proved to be less influential in overall accuracy than the scale parameter. Variations in the scale parameter can cause fairly significant changes in overall accuracy. At best the shape parameter can cause a variation of a few hundredths in overall accuracy. In the Summer dataset, adjustments in the shape parameter were responsible for improving the final accuracy from 0.51 to 0.55. With the Fall image, the highest accuracy (0.54) was achieved simply by fine-tuning the scale parameter values. The compactness-smoothness parameter did not improve the overall accuracy in either image.

### 3.4 Classification

**Figure 5.**
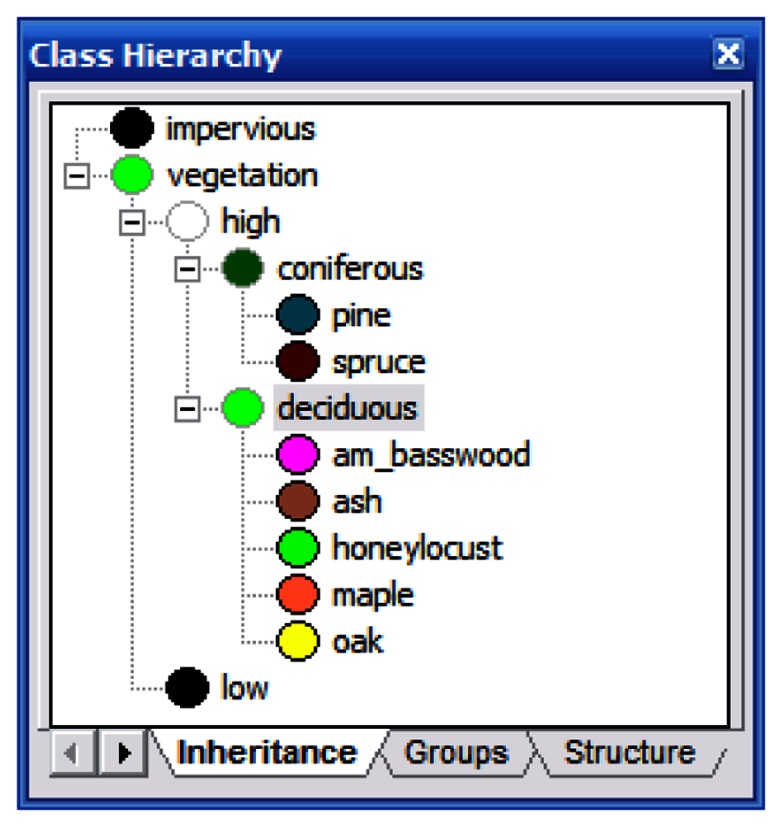
The classification hierarchy developed for LiDAR-based classifications.

Identical classifications were performed on the Summer and Fall images using the ancillary data and methods. The classification hierarchy (Figure 5) was developed as follows: First, vegetation and impervious surfaces were separated using a Normalized Difference Vegetation Index layer. The vegetation layer was subdivided into high and low vegetation using the LiDAR elevation layer. The low vegetation area represents the end of this branch of the hierarchy as we were not concerned with classifying grass and small shrubbery. The high vegetation branch was subdivided into coniferous and deciduous. This was achieved by using the LiDAR intensity layer. During visual inspection of this layer, it was readily apparent that coniferous trees reflected more in the near infrared spectrum than did the deciduous trees. The remaining classes (the tree species) were assigned to their appropriate parent classes.

The individual species were classified using a nearest neighbor classification scheme. The nearest neighbor feature space was determined using Feature Space Optimization. This tool uses samples and determines which bands are most critical to accurate classification. As important as it is to determine which bands are most important for improving accuracy, it is also important to determine which bands decrease overall accuracy and remove them. This was the case with the data in this classification. With a total of 28 bands available for the Summer image (24 MNF bands, LiDAR intensity and elevation, brightness and max difference), the ideal combination of bands excluded the MFN bands 1 and 6 as well as the Max. Dif. bands. In the case of the Fall imagery, there were 29 bands available (25 MNF bands, LiDAR intensity and elevation, brightness and max difference). Bands 4, 7, 9, 11, 15, Brightness and Max.Dif. were excluded from the classification. A number of other combinations of bands were tried including Standard Deviation, mean diff. to neighbors, and various shape attributes. These all provided poorer classification accuracy than using the MNF mean layer values. Finally, accuracy assessments were performed using the referenced data mentioned in section 3.2. A traditional confusion matrix was used to derive all accuracy statistics.

## 4. Results

**Figure 6.**
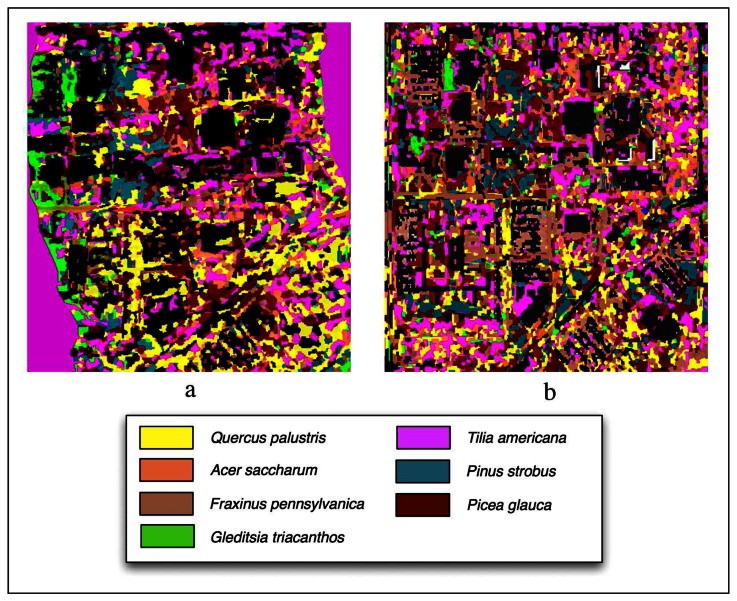
Classifications performed without LiDAR using the (a) Summer imagery and (b) Fall imagery.

Figure 6 shows classification outputs without using LiDAR for Summer and Fall season and Table 1 provides overall and class accuracies. The Summer image provided the highest overall accuracy (48%) when compared to the Fall (45%). The *Picea glauca* and *Gleditsia triacanthos* had the most robust classifications (82% producer's, 45% user's and 74% producer's, 78% user's). *Pinus strobus* (31% producer's and 38% user's) and *Acer saccharum* (25% producer's and 47% user's) had the worst accuracy.

The Fall classification had an overall accuracy of 45%. The highest accuracy belonged to *Gleditsia triacanthos* with 77% producer's and 88% user's accuracy. Lowest accuracies are held by *Pinus strobus* (19% producer's and 58% user's) and *Acer saccharum* (8% producer's and 17% user's). In comparison, the Fall data improved the accuracy of two classes - *Fraxinus pennsylvanica* and *Gleditsia triacanthos*. The Fall data is also responsible for a significant decrease in accuracy for *Acer saccharum*.

**Figure 7.**
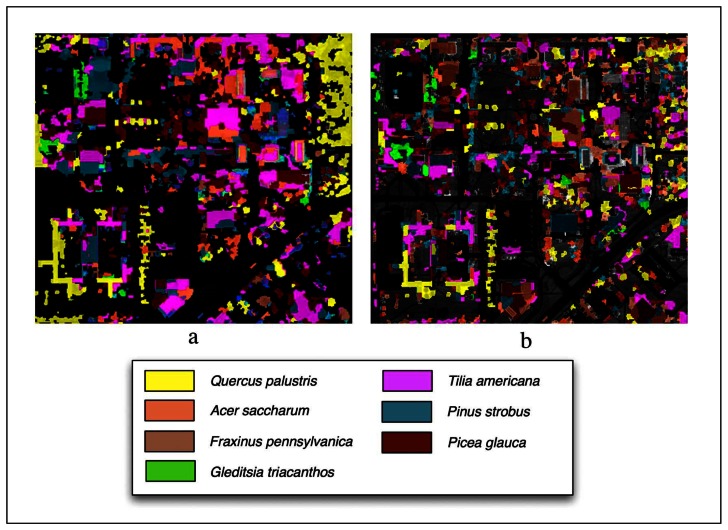
LiDAR aided object-oriented classified images using the (a) Summer imagery and (b) Fall imagery.

Classifications performed using LiDAR are shown in Figure 7 and overall and class accuracies are found in Table 2. The overall accuracy using the Summer image was 57%. The highest class accuracies were *Gleditsia triacanthos* (82% producer's, 84% user's) and *Picea glauca* (95% producer's, 59% user's). The lowest class accuracy was *Fraxinus pennsylvanica* (11% producer's, 80% user's).

The Fall image had slightly lower overall accuracy (56%). The highest accuracies were again *Picea glauca* (84% producer's, 59% user's) and *Gleditsia triacanthos* (77% producer's, 83% user's). The *Acer saccharum* class had lowest accuracy (33% producer's, 33% user's). The coniferous classes both had a decrease in accuracy. Overall, there did not seem to be a clear trend towards improvement from one season to the next. Further, the coniferous classes did not demonstrate an improvement or decline in accuracy from season to season. The *Pinus strobus* class improved from Summer to Fall while the *Picea glauca* class decreased from Summer to Fall. Alternatively, it generally appears that there is a trend toward a decrease in accuracy from Summer to Fall with deciduous species. *Fraxinus pennsylvanica* is the notable exception. This particular class showed dramatic improvement from Summer to Fall. Otherwise, accuracy was similar or decreased from Summer to Fall.

In the Summer classification, the *Picea glauca* class was very well-defined among the coniferous class as there was almost no confusion with any other classes. The *Pinus strobus* tended to be easily confused with *Picea glauca. Quercus palustris* and *Gleditsia triacanthos* also had strong classifications with little confusion among other classes. The *Acer saccharum* had significant confusion with *Tilia Americana*. The *Fraxinus pennsylvanica* class was misclassified as all other classes almost uniformly.

In the Fall classification, there was little confusion with *Picea glauca*. There was less confusion between the *Picea glauca and Pinus strobus*, and this is reflected in the improved accuracies. The *Quercus palustris* class had the most confusion with *Fraxinus pennsylvanica*. The *Tilia Americana* class had more confusion among other classes than it had with the Summer classification. The *Fraxinus pennsylvanica* class had improvements although there was confusion with *Acer saccharum. Gleditsia triacanthos* did not exhibit significant change from the Summer classification.

LiDAR generally improved the accuracy of classification when compared to classifications based on imagery alone. LiDAR improved overall accuracy 19% for both datasets. It is interesting to note that the *Fraxinus pennsylvanica* class had decreased accuracies when LiDAR was incorporated for both seasons. The class which benefitted most from LiDAR was *Quercus palustrs* in the Summer with a 55% increase in producer's accuracy and 87% in user's accuracy, and *Acer saccharum* in the Fall with a 75% increase in producer's accuracy and 50% in user's accuracy. LiDAR had very little effect on *Gleditsia triacanthos* accuracy (10% producer's increase and a 7% user's increase) in the Fall and a decrease of 6% user's accuracy in the Summer. This class is represented by trees that are grouped together, which resulted in a large segment regardless of the presence of LiDAR. One of the benefits of LiDAR is its ability to create separate segments for individual trees. With *Gleditsia triacanthos,* the segments were basically the same size regardless of the presence of LiDAR. *Fraxinus pennsylvanica* also lost accuracy with LiDAR by decreasing 21% in producer's accuracy and 19% in user's accuracy. Generally however, most class accuracies were increased by the addition of LiDAR.

## 5. Discussion and Conclusion

These results indicate that there is little difference between Summer and Fall classification accuracy when using 2m hyperspectral imagery to classify seven tree classes, In general, results from the Fall classification are more consistent. The Summer classification exhibits more extreme highs and lows with *Picea glauca* (95% producer's and 59% user's accuracies) and *Fraxinus pennsylvanica* (11% producer's and 80% user's accuracies). Some articles have indicated that September before the leaves change is the ideal time to collect imagery [1] while others have indicated that October at “peak autumn colors” provides good results [13]. However, these articles dealt with smaller numbers of classes of deciduous species. This study did not provide results to back up those conclusions. In fact, results indicate that Summer imagery provides better average accuracy for deciduous species - 51% producer's and 70% user's accuracies for Summer and 48% producer's and 55% user's accuracies for Fall. The margins are slimmer for the coniferous species averages - 71% producer's and 65% user's accuracies for Summer and 74% producer's 63% user's accuracies for Fall.

Generally, LiDAR improved overall classification accuracies by 19%. It improved producer's accuracy by an average of 19% and improved user's accuracies by an average of 31%. LiDAR can provide significant improvements in terms of both overall and class accuracies. Generally, it increased class accuracies despite a few aberrations. LiDAR provides several benefits in classifications: first, it can be used to create segments which are not influenced by shadows; second, it can be used to create class rules, such as those based on height; and third, the intensity data extend the spectrum slightly into the infrared as the laser operates at approximately 1050nm.

**Figure 8.**
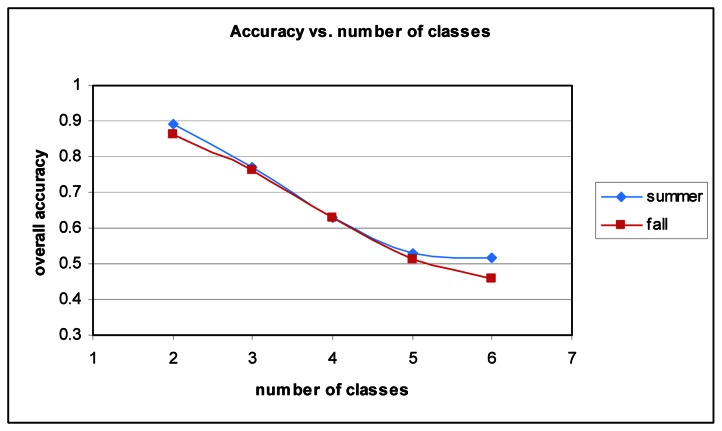
Overall accuracy vs. number of classes for Summer and Fall datasets.

While the results are as strong as they can be given the data and sample size, other researchers have achieved better results. One of the key reasons for the lower accuracy can be attributed to the number of classes. Many studies do not attempt to classify as many species. Figure 8 shows the relationship between the number of classes and overall accuracy. It stands to reason that with increased complexity classification accuracy will suffer. With two classes (*Picea glauca* and *Quercus palustris*), accuracy is approximately 89% for Summer and 86% for Fall.

Results could also be enhanced by improving the sampling method for each individual dataset. No advanced endmember selection techniques were used. The only criteria for selection were that the trees could not be close to buildings (to eliminate shadows) and that they could not be small, immature trees. The randomly-selected sites might not be ideal selections for each class. For example, random site selection could mean that a small tree was selected as a training sample even though it was in the shadows of a group of different species of trees. However, because the goal was to compare the two hyperspectral data sets, an entirely random sampling procedure was developed. Future classifications would benefit from a more selective sample generation process.

Finally, the number of samples used for the classification was below the level recommended particularly in light of the complexity of the classification [39, 40, 38]. Generally, the number of recommended training samples ranges in the hundreds. In our study, the number of training samples was limited by the number of trees of each species in the study area. By reducing the number of classes and increasing the number of training samples, overall and class accuracy would certainly increase.

## Figures and Tables

**Table 1.** Class and overall accuracies for classification performed without LiDAR for the Summer and Fall images.

Summer	*Pinus strobus*	*Picea glauca*	*Quercus palustris*	*Acer saccharum*	*Tilia americana*	*Fraxinus pennsylvanica*	*Gleditsia triacanthos*
Producer's	31%	82%	49%	25%	41%	31%	74%
User's	38%	45%	36%	47%	50%	52%	78%
Overall Accuracy	48%						

Fall	*Pinus strobus*	*Picea glauca*	*Quercus palustris*	*Acer saccharum*	*Tilia americana*	*Fraxinus pennsylvanica*	*Gleditsia triacanthos*
Producer's	19%	74%	43%	8%	38%	53%	77%
User's	58%	33%	48%	17%	35%	56%	88%
Overall Accuracy	45%						

**Table 2.** Class and overall accuracies for classification performed with LiDAR for the Summer and Fall images.

Summer	*Pinus strobus*	*Picea glauca*	*Quercus palustris*	*Acer saccharum*	*Tilia americana*	*Fraxinus pennsylvanica*	*Gleditsia triacanthos*
Producer's	47%	95%	71%	31%	59%	11%	82%
User's	71%	59%	68%	58%	62%	80%	84%
**Overall Accuracy**	**57%**						

Fall	*Pinus strobus*	*Picea glauca*	*Quercus palustris*	*Acer saccharum*	*Tilia americana*	*Fraxinus pennsylvanica*	*Gleditsia triacanthos*
Producer's	64%	84%	57%	33%	33%	42%	77%
User's	68%	59%	57%	33%	54%	45%	83%
**Overall Accuracy**	**56%**						
